# Marketing sonified fragrance: Designing soundscapes for scent

**DOI:** 10.1177/20416695241259714

**Published:** 2024-08-02

**Authors:** Charles Spence, Nicola Di Stefano, Felipe Reinoso-Carvalho, Carlos Velasco

**Affiliations:** Crossmodal Research Laboratory, 150583 Department of Experimental Psychology, University of Oxford, Oxford, UK; Institute of Cognitive Sciences and Technologies, National Research Council of Italy (CNR), Rome, Italy; School of Management, Universidad de los Andes, Bogotá, Colombia; Centre for Multisensory Marketing, Department of Marketing, BI Norwegian Business School, Oslo, Norway

**Keywords:** sensory marketing, crossmodal correspondences, audio branding, scent, fragrance marketing

## Abstract

Auditory branding is undoubtedly becoming more important across a range of sectors. One area, in particular, that has recently seen significant growth concerns the introduction of music and soundscapes that have been specifically designed to match a particular scent (what one might think of as “audio scents” or “sonic scents”). This represents an exciting new approach to the sensory marketing of fragrance and for industries with strategic sensory goals, such as cosmetics. Crucially, techniques such as the semantic differential technique, as well as the emerging literature on crossmodal correspondences, offer both a mechanistic understanding of, and a practical framework for, those wishing to rigorously align the connotative meaning and conceptual/emotional/sensory associations of sound and scent. These developments have enabled those working in the creative industries to start moving beyond previously popular approaches to matching, or translating between the senses, that were traditionally often based on the idiosyncratic phenomenon of synaesthesia, toward a more scientific approach while nevertheless still enabling/requiring a healthy dose of artistic inspiration. In this narrative historical review, we highlight the various approaches to the systematic matching of sound with scent and review the various marketing activations that have appeared in this space recently.

There is, as it were, an octave of odours like an octave in music; certain odours coincide, like the keys of an instrument. (G. W. Septimus Piesse, The Art of Perfumery, 1867)

## Introduction

There has been a rapid growth of interest in the field of auditory branding in recent years (see Spence & Keller, 2024, for a review). One area that has become popular is the introduction of music and soundscapes that have been specifically designed to match a particular scent (what one might think of as “audio scents” or “sonic scents”). In this narrative historical review (see Ferrari, 2015; Furley & Goldschmied, 2021, on the strengths and appropriateness of this style of review), the various approaches to the systematic matching of sound and scent that have been used are highlighted. Additionally, various recent marketing activations in this space are also discussed. The review calls attention to how techniques such as the semantic differential technique, as well as the emerging literature on crossmodal correspondences, offer both a mechanistic understanding of, and a practical framework for, those wishing to align the connotative meaning and conceptual/emotional/sensory associations of sound and scent. Such conceptual frameworks have enabled those working in the creative industries to move beyond previously popular approaches to the matching or translation between the senses that were often based on the idiosyncratic phenomenon of synaesthesia, toward a more scientific approach while nevertheless still enabling/requiring a healthy dose of artistic inspiration.

This manuscript explores the dynamic relationship between sound and scent, investigating how corresponding sensory experiences can be systematically aligned for enhanced perception in both commercial and digital contexts. We start with an introduction to the psychological underpinnings of sound–scent associations, followed by a discussion on commercializing musical perfumes. The core of the review systematically examines the alignment of sound and scent through various methodologies, including the semantic differential technique and crossmodal correspondences, while also addressing the challenges and philosophical considerations involved. The manuscript concludes by summarizing the insights gained and their implications for future research and practical applications. Ultimately, the primary focus is on the sonic design of soundscapes to match pre-existing fragrances and the aromas/flavors of specific food and drink products. That said, the occasional attempts to design scents for sounds are also mentioned briefly.

Sound has become one of luxury's most powerful tools; so read the headline of an article that appeared in the Robb Report a couple of years ago (see Alexander, 2021). Indeed, there can be little doubting that the world of sonic branding/brand identity has become increasingly important in the contexts of both academic and industry in recent years (e.g., see Knoeferle & Spence, 2021; Taylor, 2023a, b; Vidal-Mestre et al., 2022). Interestingly, while fragrance and cosmetics brands have long invested heavily on crafting their visual identities (Schifferstein & Howell, 2015; Schifferstein & Tanudjaja, 2004; Spence, 2020a, b), marketing in this sector (e.g., Bradford & Desrochers, 2009; Brady, 1978; De Luca & Botelho, 2021; Minsky et al., 2018)^1^ has only recently really started to move in the direction of linking scent with specially designed sonic assets (Errajaa et al., 2023; IFF, 2023; Mensing, 2023; Rodriguez, Arboleda et al., submitted; see also Carnevale et al., 2023),^2^ with some famous pop stars directly contributing to the market by creating their own fragrance collections (e.g., see Katy Perry).^3^ In fact, very recently, International Flavors & Fragrances (IFF) launched a sound logo to illustrate multisensory dimensions of scent: Named “World of Scents,” the sound is supposed to represent “how scent plays a key role to awaken the extraordinary senses” (IFF, 2023).^4^

One reason to develop sonic assets that, in some meaningful sense, match a fragrance, can be framed in terms of the emerging field of sensory (or better said, multisensory) marketing (Knoeferle & Spence, 2021; Velasco & Spence, 2019). Indeed, many practitioners are becoming increasingly interested in facilitating/enhancing their online marketing activities by capitalizing on the senses (e.g., Mahdavi et al., 2020). After all, the senses play a key role in the experience economy (Pine & Gilmore, 1998), with many premium products and services differentiating themselves as a function of the experiences that they happen to deliver. The meaningful integration of sight, sound, touch, taste, and smell^5^ has been shown to enhance the creation of memorable and authentic consumer experiences across a range of industries (Eglīte, 2021).

A multisensory approach is also crucial to the design and creation of novel products, including fragrances. For example, according to Mensing (2023, p. 225): “The quickest and most effective way to achieve new inspiration is to fuse the fragrance experience with other sensory impressions, so that the fragrance is supported by colour, form, music, and ideally also tactile and gustatory.” Indeed, most everyday experiences are multisensory in nature, thus highlighting the importance of considering the integrated impact of multiple human senses on the experiences of consumers, no matter where that might be occurring (Spence, 2021c; Velasco & Obrist, 2020; Velasco & Spence, 2019). Scents and sounds, more particularly, are crucial to consumer experiences given their putatively unique ability to evoke emotions, enhance product recall, and create a distinctive atmosphere, among others (e.g., Spence, 2021a).

For instance, in one early multisensory study on audio–olfactory interactions, Spangenberg et al. (2005) investigated the effects of combining an ambient Christmas scent (“Enchanted Christmas”) and Christmas-style music on North American consumers’ perception and evaluation of the products that were displayed in a mock retail store. The presence of Christmas music (Amy Grant's “Home for Christmas,” 1992) enhanced favorable evaluations when paired with a Christmas scent whereas music from the same artist that was not associated with Christmas diminished the participants’ evaluations. These results highlight the moderating role of background music in multisensory olfactory and musical stimuli interactions (at least in the context of a mock retail store). In summary, Spangenberg et al.'s results show that the semantic congruency (i.e., aligning sensory inputs as a function of a common identity or shared meaning, such as Christmas) between ambient scent and music led to a more favorable evaluation of the store, its merchandise, and the store environment.

Combining olfactory stimuli with sound might well also be expected to affect the purchase experience of perfumes more indirectly. Think only of how music can be used to modulate consumers’ mood/emotions when choosing fragrance, or to identify potentially liked fragrances among the ones that people with similar musical preferences tend to appreciate. Recently, Mensing (2023, p. 324) highlighted the relationship between musical preferences and contemporary trends in fragrance marketing, stating that: “Changes in style can be seen first in fashion and music before they are reflected in fragrance trends. As a trend coach for perfumers, I pay special attention to change, i.e., the increasing or decreasing acceptance of music genres.” What is more, new crossmodal approaches to fragrance shopping suggest that an individual's musical preferences may well be in some meaningful way related to their preferences for particular perfumes (see below for one example of a playful approach to sound–scent matching from https://www.fragrancedirect.co.uk/blog/fragrance/find-your-sound-with-perfume-and-music/).^6^ It is, though, an open question as to whether such crossmodal prediction represents a literal example of sensory translation (see Spence & Di Stefano, 2023, on this theme), or rather it should be considered as an example of associative learning.^7^

The Scent: Armani My Way

The Sound: Masego - Silver Tongue Devil

Got a tropical holiday booked? First, you’re going to want to purchase this gorgeous fragrance from Armani for its stunning fruity notes of bergamot, orange blossom, tuberose and white musk. Second, you need to add this island-vibe track to your holiday playlist pronto! From the first few seconds of the song, you’ll be hooked and picturing yourself sipping cocktails on a sun-lounger before you know it. Masego is a musical genius, fusing his saxophone melodies into many of his iconic tunes.

Importantly, shoppers are not able to smell the scents marketed online. However, a perfume brand can certainly play the appropriate jingle (perhaps heard via computer or smartphone), which may help to guide the shopper to the right scent for them (cf. Knoeferle et al., 2016; Mensing, 2023). Such developments have become crucial in recent years given the continued problems/challenges associated with the digitization of scent delivery (see Spence et al., 2017), for example, as part of digital sensory marketing campaigns (Petit et al., 2019). Looking to the future, certain marketers promise to blend online and offline through mixed reality involving olfactory auditory, and visual experiences (perhaps also incorporating the other senses as well). The integration of sound and smell in digital technology experiences, explored by academics such as Obrist et al. (2017) and Kerruish (2019) in the context of new sensory-enabling technologies has already found application in the context of virtual reality (VR), where the importance of aroma–content congruence is emphasized (Flavián et al., 2021).

### Psychological Mechanisms Explaining the Links Between Sound and Scent

**Figure 1.**
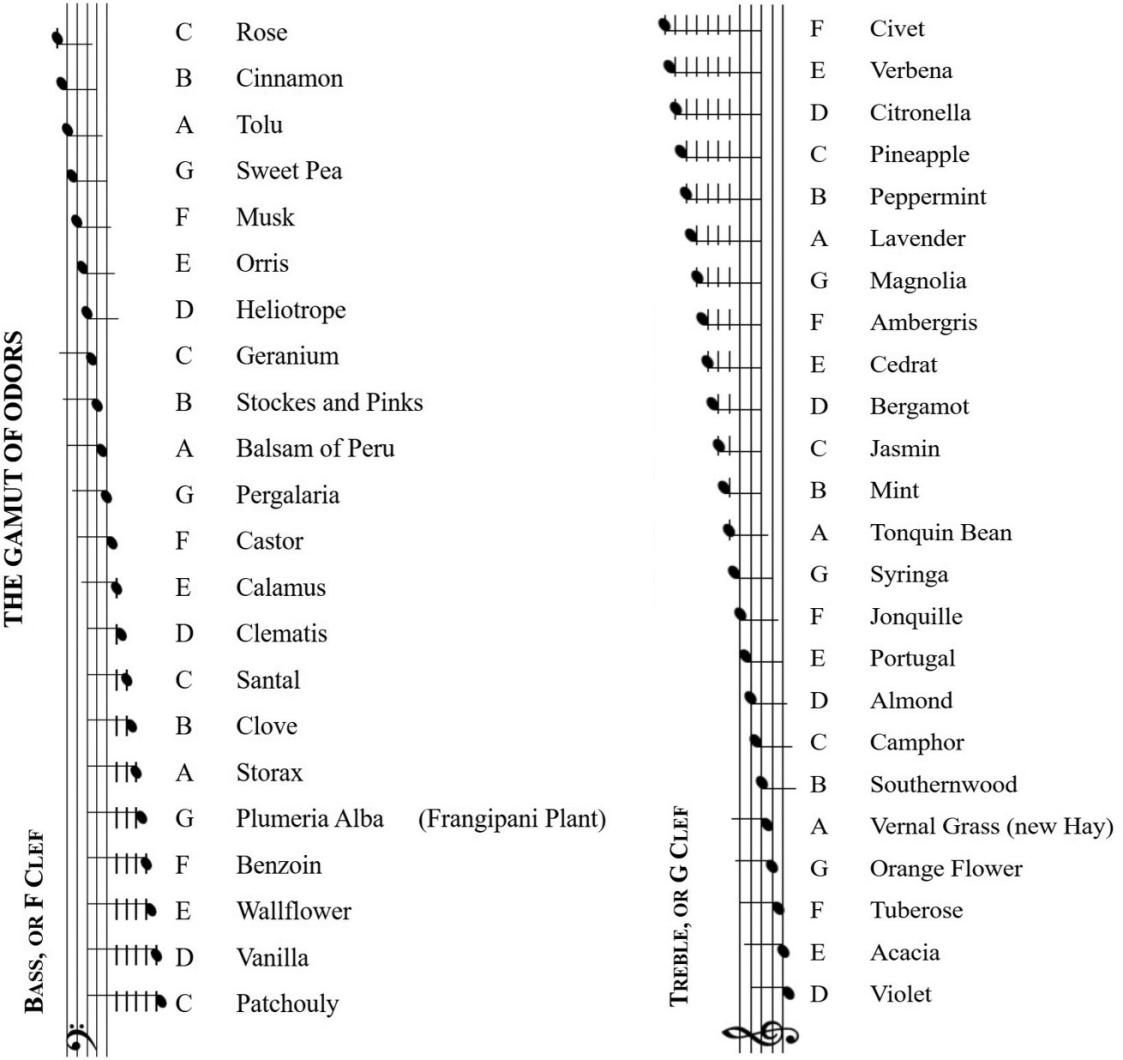
Crossmodal correspondences between musical notes and odors, as suggested by Septimus Piesse (1867, pp. 42–43), are illustrated on a scale.

There is currently growing academic interest in researchers exploring the links between fragrance and sound, connecting back to the early chemist, Septimus Piesse's “Gamut of Odors” (Piesse, 1867, see Figure 1). The experimental psychology and cognitive neuroscience literature here both provide useful frameworks when it comes to thinking about how best to match sound, soundscapes,^8^ and music to scent. Perhaps the most obvious approach to connecting the two senses relies on the semantic congruence approach described above in the study of Spangenberg et al. (2005), whereby smell and sound are aligned in terms of a given identity or meaning, and therefore both denote said identity or meaning (e.g., the smell of Christmas and the sound of Christmas).

Perhaps less direct, another approach, the semantic differential technique (Kawachi et al., 2011; Oyama et al., 1998; Snider & Osgood, 1969), can be used to assess the key connotative (implied) meanings that people associate with fragrances (Dalton et al., 2008) as well as with music (Watt & Quinn, 2007; though see also Miller, 2021).^9^ The semantic differential technique is a tool characterized by its use of bipolar pairs of adjective (e.g., nice-awful, mild-harsh, and powerless-powerful) to help quantitatively measure the semantic meaning attributed by individuals to concepts or objects, thereby facilitating the assessment of the said connotative meanings, which are broadly categorized (often as the result of multi-dimensional scaling) along three principal dimensions: evaluation, potency, and activity (Osgood et al., 1957).

**Figure 2.**
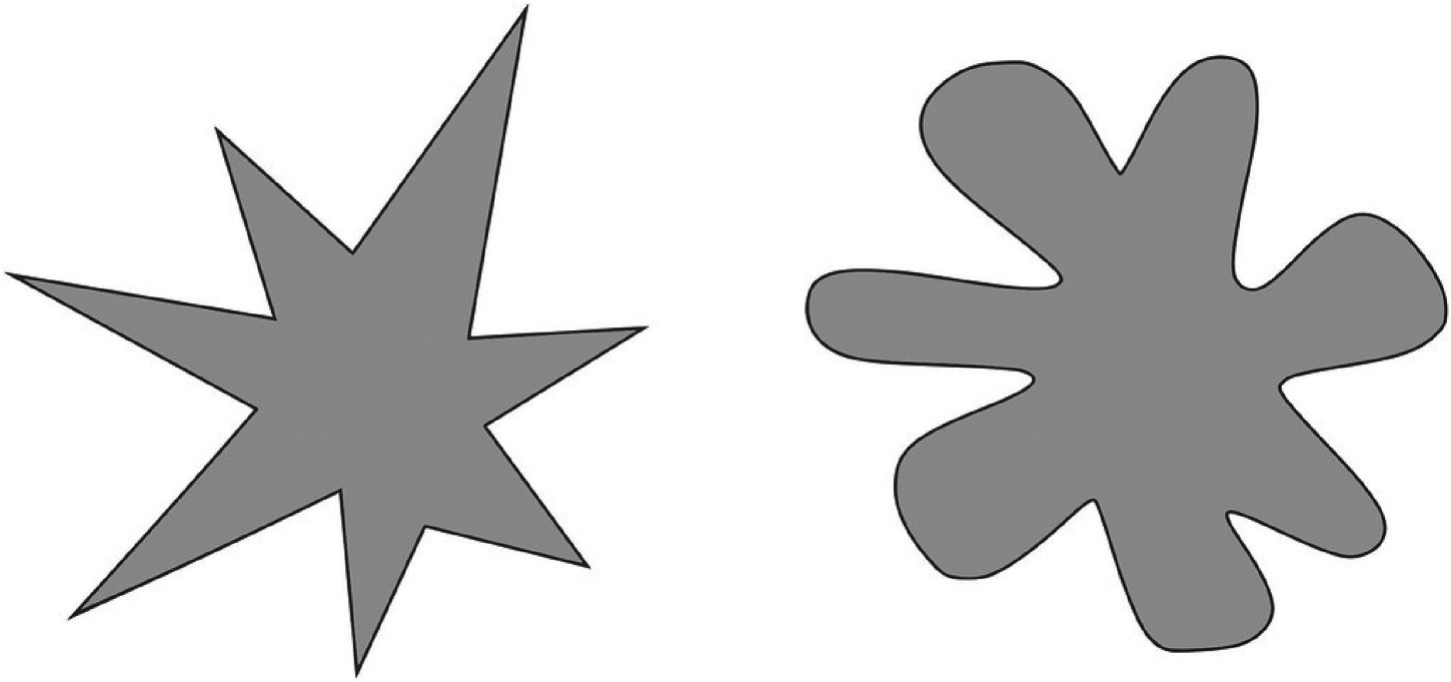
What do these shapes smell like? Which of the two would you associate with a sweet smell?

At the same time, however, the emerging literature on the crossmodal correspondences (Spence, 2011) provides an alternative framework when it comes to thinking about the multitude of connections that exist between features across the senses (that, crucially, does not rely on synaesthesia).^10^ For example, the literature on the crossmodal correspondences shows that most people will tend to associate the colour pink and round shapes with the taste of sweetness (Velasco et al., 2023b). What is more, crossmodal correspondences are typically transitive (meaning that if a sensory characteristic A corresponds with another B, and B corresponds with C, then A would likely correspond with C, as well). So, for example, if pink happens to be associated with sweetness, and round is also associated with sweetness, then pink should be associated with round (Deroy & Spence, 2013c). At the same time, however, a number of crossmodal correspondences between sound and scent have also now been documented (see Deroy et al., 2013). For instance, people have been shown to associate those smells described as sweet with round shapes (Hanson-Vaux et al., 2013, see Figure 2). Moreover, fruity odors are associated with high-pitched musical notes (Crisinel & Spence, 2012), while the aroma of orange and iris flower is linked to pitches that are significantly higher than those associated with the scents of musk and roasted coffee (Crisinel & Spence, 2012).

Interestingly, crossmodal correspondences have been classified as a function of a series of nonmutually exclusive underlying mechanisms: These include structural, statistical, linguistic, and affective correspondences (Motoki et al., 2023; Spence, 2011; see also Ohtake et al., 2023), which can help guide the selection of congruent and incongruent sound/smell combinations to guide specific experiences involving these senses. According to Spence (2011), structural correspondences involve similarities in the neural coding in different sensory representations, such as, for example, stimulus intensity (cf. Stevens, 1957, 1971). Statistical correspondences, by contrast, are thought to arise from the internalization of the regularities of the environment (such as high-pitched sounds tending to come from higher locations in space; e.g., Parise et al., 2014). Linguistic correspondences encompass metaphorical associations based on shared linguistic descriptors (such as the word “high” being used to describe both top notes in fragrance and high-pitched sounds), while affective correspondences stem from the shared emotional associations of various stimuli, such as the link between the taste of sweetness and shape attributes like curvature, given that both are positively valenced (Spence, 2020d).

However, one of the relevant recent changes in terms of the approach to the crossmodal alignment of scent and sound, at least compared to what has been seen previously (Spence, 2021a), is that while a series of scents would typically have been released to accompany a musical or other (e.g., dance) performance, what is happening more frequently nowadays is that an individual scent is being paired with a temporally evolving soundscape or piece of music.

## Commercializing Musical Perfumes: Soundscapes Designed to Match Specific Scents

Over the last few years, several perfumers and larger fragrance manufacturers have started to work on innovatively incorporating a sonic element into their promotional strategies (see Mensing, 2023). For example, the fragrance Spicebomb Infrared by Viktor & Rolf (in collaboration with L’Oréal Luxe Division) featured an original soundtrack created by Ircam Amplify. As specified by the press coverage accompanying this activation, sounds were selected that were supposed to convey the perfume's hot and spicy notes (Edelson, 2021; L’Oréal Group, 2021). According to Edelson (2021): “The sound IRCAM Amplify sound designer Romain Barthélémy created with IFF's Christophe Hérault ‘is a combination of all the sounds that bring the same kind of atmosphere. It's a mix of creative sound and ambient sound, like you’d hear in a nightclub or on the street. We mix all that and create the specific sound for the specific scent’.” The suggestion was that “The beauty of what it allows brands to do in the future is establish the universal correlation between the world of scents and the world of sounds… We believe it constitutes a real revolution for e-commerce and perfume sampling. Working with a sound that generates the same sensation as the original scent means you can eliminate the hurdle of not being able to literally smell the scent because you can now hear the fragrance.”^11^ Earlier this year, IFF signed a partnership with IRCAM Amplify, exploring the emotional connections between sound and scent. Together, they conducted a ground-breaking study showing that a purposefully designed sound increased scent purchase intent by nearly 60 percent in an e-commerce site.^12^

Another fragrance brand, Room 1015, known for its nostalgic fragrances, inspired by the rock ‘n’ roll era, has crafted a unique approach to perfumery. They have created a soundtrack to match for each of its fragrances, combining music and scent (ROOM 1015, n.d.). Meanwhile, L’Orchestre Parfum offers a range of fragrances inspired by music, providing a dedicated soundtrack for each perfume (L’Orchestre Parfum, 2021). The emphasis, though, in the case of Jo Burzynska's perfume, Eau Tautahi, which was released together with a paired urban soundscape (Papesch, 2022), would appear to be very much on the fragrance, with the soundscape designed to complement the scent rather than vice versa. Burzynska is a sound artist and professional wine judge who created both the scent and, thereafter, the matching soundscape (see Burzynska, 2018).

Developments in the pairing of scent with sound have, though, also come from the other direction. The singer Arooj Aftab, for instance, released a fragrance to accompany her album Purple Vulture back in 2019 (Jagota, 2021). In the latter case, the primary component in the combination would appear to be the music. Meanwhile, in the case of pop stars, such as Katy Perry, who have started to create fragrances, one might question whether they should in any meaningful sense represent the same stylistic features of their music. Meanwhile, Robin Fox, an Australian musician (https://en.wikipedia.org/wiki/Robin_Fox_(Australian_musician)), created a set of fragrances that were linked to his music in 2015 (see Robin, 2015). It is worth noting that customers may respond differently were they to be informed that the music served as the source of inspiration for the creation of the fragrance^13^ or vice versa (cf. Reinoso Carvalho et al., 2015). As of yet, we are unaware of any examples where the scent and sound have actually been developed in parallel, if indeed such a creative process is even possible. Yet, this is a potentially interesting approach whereby any marketing concept might, from the start, consider how different sensory cues might be part of a cohesive storytelling strategy (cf. Velasco et al., 2018).

The interest in combining sounds with scent can also be seen in the world of fragranced Home and Personal Care products (e.g., just take the following quote from a 2007 marketing campaign: “*The marketing team captured the idea of appealing to the senses with lots of colour, music, perfumes & energy*,*”* Marco Luconi, Regional Brand Manager, Latin America, for an early attempt to match the music to the scent).^14^ While the above examples clearly demonstrate the interest in developing marketing strategies that are based on the combination of sound and fragrance, it seems that the criteria that have been used to incorporate sonic elements into promotional strategies of fragrances are based on the individual taste or the aesthetic/artistic view of creators. However, one could ask whether there are other ways in which to combine sound and fragrance that might be based on more objective, or at least intersubjective, criteria, and that might therefore lead to marketing campaigns that are potentially more effective in terms of communicating with, and/or engaging, the consumer. In the next section, we address this important question by examining the semantic differential technique, crossmodal correspondences, and the phenomenon of sensation transference.

## Systematically Aligning (the Meaning of) Sound and Scent

Several techniques can potentially be used to help establish the connotative (not to mention the denotative) meanings of, and the crossmodal associations that can be attached with, specific sensory stimuli, such as, for example, scents and sounds. These include the semantic differential technique and the emerging literature on the crossmodal correspondences.

### The Semantic Differential Technique

**Figure 3.**
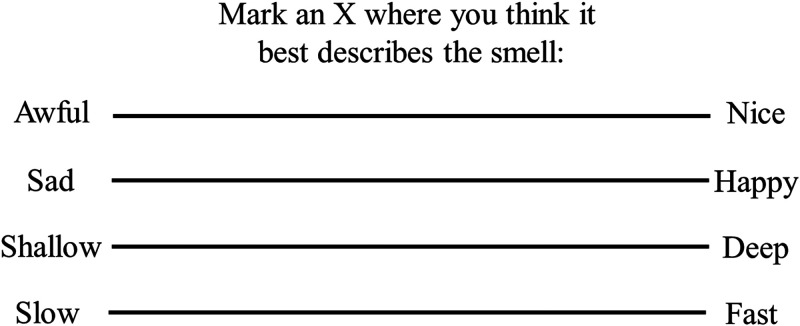
Example of the kind of semantic differential scales that have been used in previous research.

Importantly, recent years have seen a move away from the synaesthetic approach to translating between the senses (Haverkamp, 2014) that may well have stymied research unnecessarily (Spence, 2012, 2015a).^15^ Previously, researchers have used semantic differential scaling (e.g., Osgood et al., 1957; Oyama et al., 1998) in order to help define the multidimensional representation (or connotative meaning) of odours (Dalton et al., 2008). Crucially, the semantic differential technique has also been applied to music perception as well (e.g., Hacohen & Wagner, 1997; Holbrook & Huber, 1979; Miller, 2021; Scherer & Oshinsky, 1977; Windsor, 2004). Typically, the multidimensional scaling of people's responses to a range of bipolar attribute scales (viz., good-bad; strong-weak; and rough-smooth, see Figure 3, for an example) is used to help reduce complex stimuli or concepts down to three principal dimensions of meaning, namely Pleasure, Arousal, and Dominance (see, e.g., Di Stefano et al., 2024).

While the administration of the semantic differential instrument is not in itself too challenging, the interpretation of the results (especially in the context of musical meaning) is a very different matter (Miller, 2021; see also Auracher et al., 2020). It can also be difficult to falsify the idea that sensory attributes can be expressed in terms of more or less underlying bipolar dimensions. In other words, when asked to evaluate a sensory characteristic (e.g., a smell or a sound) in terms of polar dimensions, it is likely that some sort of connotative meaning will always be implied. As such, it might be necessary to experimentally manipulate the different sensory characteristics in relation to a given connotative meaning in order to determine the extent to which they actually have a given meaning, one that the semantic differential technique might not necessarily capture (Woods et al., 2013). It is perhaps for these reasons that Saitis et al. (2020) advocated that there should be a movement beyond the traditional use of the semantic differential method for collecting behavioral data and toward the study of crossmodal correspondences between timbral dimensions and other sensory modalities (see also Di Stefano, 2023). At a practical level, though, the semantic differential technique might offer practitioners a way to align sensory cues in a meaningful manner.

The collaboration between scent and sound designers that gave rise to Viktor & Rolf's Spicebomb Infrared would appear to have been based on a somewhat similar approach. Writing about the collaboration, Edelson (2021) notes that “The methodology, developed internally by IRCAM, is called SPEAK, which helped the two teams find a common language. ‘Speak is a software toolbox to help people communicate when they're from two different worlds and trying to find the words,’ Tour said. ‘It's based on psycho-acoustic research based on how the brand interprets sounds. We put that in those tools, and we have a kind of semantic language: sound. We worked together to put words on something like a very subjective sound or flavour. After a couple of iterations, we came to the conclusion that it's the right message and the right sound’.” It would be interesting to know more about how SPEAK is related to approaches such as that of the Semantic Differential Technique (see https://support.ircam.fr/docs/speak/ for more information on the SPEAK approach).

### Crossmodal Correspondences Between Scent and Sound

Many of the recent developments creatively connecting sound and scent have been built on the emerging literature on the crossmodal correspondences that have increasingly been documented between scents, sounds, and a host of other sensory features (e.g., Deroy et al., 2013; Di Stefano et al., 2022; Hanson-Vaux et al., 2013; Mesz et al., 2023; Rodriguez et al., 2023a; Saitis et al., 2020; Wallmark, 2019). As such, it suddenly becomes meaningful to ask: “What does a sweet and a dry smell sound like?” In fact, there has been a veritable explosion of research in “sonic seasoning” (Spence, 2017; see also Klaverstijn, 2021), that is, the matching of music to the flavor of food and drink (see Spence et al., 2019). Given that something like 75–95% of what we think we taste we actually smell (Spence, 2015b), it is obviously natural to extend the approach from matching sounds to flavors and food aromas, to the matching of soundscapes to fragrance.

Researchers have previously identified an association between both perfumery notes (Belkin et al., 1997) and selected wine-related aromas (Crisinel & Spence, 2012; see also Piesse, 1867). However, as noted recently by Zacharakis et al. (2023), the majority of the evidence that has been published to date has tended to focus on pitch-aroma correspondences with timbral-aroma relationships being largely associated with specific sound sources (i.e., classes of musical instrument, such as, for instance, wind or strings)^16^. Several studies conducted over the last decade or so have investigated the way in which specific scents are matched to basic musical features. Bronner et al. (2012) conducted a series of two experiments: in the first of which participants tasted or imagined two flavors (“orange” and “vanilla”),^17^ and performed several crossmodal associations and matching tasks. In the second study, meanwhile, short auditory clips were specially designed to yield different citrus-like sounds. Based on a matching task, their results showed that “orange” evoked bright, fairly sharp, and rough timber, staccato, accentuated, dynamic articulation, syncopated rhythm, medium to large intervals melody, mid-to-large range ambitus, and lively and fast tempo musical parameters. In contrast, “vanilla” evoked soft and dull timbre, legato, even, minimal dynamic articulation, even rhythm, small intervals and consonant melody, small range ambitus, and rather slow tempo. While these descriptors also refer to flavors, they are also dominant notes in the context of perfumery. Their results showed that manipulations along the weak–strong and acute–dull axes of intensity and roughness/brightness caused corresponding perceptions of the stimuli across the gustatory and auditory modalities.

Meanwhile, Cantu et al. (2022) conducted a study to determine sound qualities that matched freshness. These researchers conducted two online experiments in which the participants rated sensations and odor notes while listening to sounds. In this case, the results revealed that sounds in which predominant musical features included woodwind and piano instruments, low tempo, and legato articulation were able to trigger sensations and olfactory notes associated with freshness, such as humidity, outdoors, lightness, smoothness, coolness, calmness, freshness, and fruitiness, while avoiding the recall for spiciness (opposite to freshness). The same sounds were mainly associated with green and blue colors, which are usually matched with freshness.

Zacharakis et al. (2023) recently conducted a study designed to assess the strength of the crossmodal correspondence between 12 odors and 26 complex musical tones that had been intuitively chosen/created in order to match a range of odors. The olfactory stimuli consisted of vanilla, honey, caramel, cinnamon, coffee, (black) pepper, lemon, lemon blossom, pomegranate, melon cherry, and tobacco. According to the study authors, “A particular instruction was also given to ignore any conscious higher-level connections between sounds and concepts related to the source of the aromas (e.g., ‘This sounds like a buzzing bee therefore it has to be associated with honey’) but to base judgements merely on potential sensory associations as much as possible.”^18^ A group of 29 musically trained participants listened to the musical stimuli in a random order. They had to try and associate each sound with one of the olfactory stimuli (note that an aromatic label was provided next to each scent). The results highlighted the existence of crossmodal associations that were above chance for 20 out of the 26 synthesized sound stimuli and at least one of the 12 provided olfactory stimuli.^19^ These intriguing results therefore suggest that it is indeed possible to obtain reliable crossmodal associations (or matches) between complex timbres and selected aromas.

Another point to consider here is that the consensual crossmodal and conceptual associations of sounds and scents that have been highlighted in the literature on the crossmodal correspondences and the semantic differential technique are potentially different in kind from the meaning that is sometimes attached by the listener to music that has been intentionally designed (see Miller, 2021, on this theme; cf. Watt & Ash, 1998). As such, it may be difficult to know for certain the level at which such crossmodal matching should take place. To further this discussion, incorporating both explicit—that is, the stereotypical self-report—and implicit—that is, the Implicit Association Test (IAT)—measures during such assessments might prove beneficial (Rodriguez, Cantu et al., submitted). Implicit measurements primarily seek to identify underlying automatic associations, reflecting the system's responses to unconscious situations, effortlessly and without self-control (Greenwald et al., 1998). This approach allows the gathering of information about how target stimuli are associated, without explicitly instructing individuals to be aware of crossmodal correspondences (Demattè et al., 2007). In summary, implicit and explicit mechanisms evoke distinct consumer responses, not always yielding outcomes that are positively correlated (Gawronski & Hahn, 2018).

### Challenges to the Matching of Scent and Sound

The language of scent and sound often overlap with similar descriptors such as top notes, bass notes, chords, harmonies, and accords being used to describe the stimuli in both modalities (see also Piesse, 1867). Moreover, speakers also often refer to both odors and sounds by mentioning their sources (Barwich, 2019; Nudds, 2010). At the same time, however, there are also a number of fundamental differences in terms of the relation between stimulus and perception in the two senses. In particular, it should be noted that there is little obvious alignment between chemical complexity and perceived complexity in the case of olfactory (or chemosensory) stimulation (e.g., Spence & Wang, 2018) whereas the link would appear to be much more apparent in the case of audition. Furthermore, the fact that scent evolves over time presumably means that any sonic composition designed to match such a scent would also need to progress temporally (cf. Mahieu et al., 2019). Here, though, another potentially important challenge arises, namely that the temporal rate of information processing in olfaction is simply much lower than it is in audition (see Gallace et al., 2012). Finally, it should be noted that researchers have long questioned whether it is even possible to experience any kind of perceptual similarity between the impressions that are available to the different senses (see Di Stefano & Spence, 2023). As such, the best that might ultimately be possible in terms of crossmodal matching is some kind of affective alignment of emotional sequences of sensations attached with the various notes and scents.

One other issue to consider relates to individual differences in crossmodal correspondences (Spence, 2022b). That is, there are undoubtedly going to be individual differences in, say, liking for individual sensory characteristics, smell thresholds, etc., as well as cultural differences in exposure to, and the meaning of, scent. So there lies an opportunity for personalization and customization. As research focuses on group level matching, there may be an opportunity here to consider individual differences in research and in practices (cf. Reinoso-Carvalho et al., 2017).

### Auditory and Olfactory Objects in the Philosophy of Perception

The nature of auditory and olfactory objects has also recently attracted a growing body of interest from those scholars working in the philosophy of perception (e.g., Barwich, 2019; Millar, 2019; Nudds, 2010; Skrzypulec, 2019). One general question that is typically raised in the current debate deals with the puzzling nature of those entities that are referred to when people talk about “odours” and “sounds”. Compared to visual objects—often considered as the prototypical perceptual objects in the Western visuocentric approach (Barwich, 2019)—odors and sounds would appear to lack key properties that should constitute perceptual objecthood, such as figure-ground segregation and perceptual constancies (e.g., Barwich, 2019, though see Millar, 2019).

A common feature of sounds and odors is that speakers normally identify them indirectly, that is, by referring to their source, saying, for example, the sound of a violin or the smell of a rose. This would appear to reveal some fundamental properties of olfactory objects given that we can perceive the smell of the rose or jasmine in the absence of the source object as happens normally in the case of fragrances. Something similar might happen with sounds, but not, at least in normal perception, with visual objects: That is, the visual perception of a ship is not veridical if the object “ship” is absent. In contrast, the perception of the scent of a rose is veridical even if the rose itself is absent. The abovementioned considerations would seem to hamper any attempt to establish a consensual crossmodal match between odors and sounds based on the structural properties of the perceptual objects. Indeed, the impermanent and evanescent nature of odors and, to some extent, of sounds, make it hard for designers and creators to solidly ground the cross-sensory matching between fragrances and music on the sensory translation between structural features of the stimuli which evolve over time (see Spence & Di Stefano, 2023). Hence, alternative strategies should be developed, likely based on other kinds of crossmodal correspondences, such as those that are statistically, affectively, or semantically mediated (Motoki et al., 2023; Spence, 2011; Spence & Di Stefano, 2023).

### Sensation Transference

“Sensation transference” constitutes one of the routes by which sonic attributes add value to scent. More specifically, a number of studies now show that the more the customer likes the soundtrack, jingle, or sonic logo, the more they will like the scent that happens to be associated with it (Spence, 2021c). So, for example, in related research on the topic of sonic seasoning, Reinoso-Carvalho and his colleagues showned that such hedonic transfer effects from soundscape to taste evaluation (in the case of chocolate samples) was more marked than any sonic seasoning effect leading to a change in the perceived sweetness/bitterness (Reinoso-Carvalho et al., 2020a; Reinoso-Carvalho et al., 2020b). Note that it is at least conceptually possible to separate the sensation transference associated with the affective value of the sonic and scented stimuli from the priming of sensory, conceptual, and higher-level musical associations of more complex stimuli.

## Crossmodal Effects of Sound on Scent

In recent years, several fragrances have been released with accompanying music tracks. Researchers have been able to demonstrate how specific aspects of music can be used to accentuate different notes in a fine fragrance. But what impact does one sense have on people's perception and evaluation of the associated stimuli presented in the other sensory modality? Relevant here, Velasco and Spence (2022) recently conducted a study that assessed the influence of soundscapes characterized as either “dry” or “sweet” on the perceived dryness and sweetness of a fragrance, measured on a continuous scale anchored with these concepts.^20^ They found that the fragrances were perceived as smelling significantly sweeter when they were accompanied by a sweet soundscape as compared to a dry one. Meanwhile, Velasco et al. (2014) investigated how the hedonic congruence between scent and sound stimuli influences participants’ perception of odor intensity, pleasantness, and quality. In this case, the results revealed that, contrary to expectations, broadband white noise had a more significant impact on participants’ odor ratings than musical selections, indicating a close relationship between an odor's hedonic character and its perceived quality. Participants rated the different scents used as less pleasant and less sweet when exposed to white noise, in contrast to any of the other musical conditions, varying in valence.

Though much of the research that has been published to date has attempted to assess the effect of scent on people's enjoyment of music (e.g., Zhou & Yamanaka, 2018), synergizing the senses, based on the emerging understanding of the crossmodal correspondences between scent, sound, and color looks set to continue growing in the years ahead, and may one day revolutionize the multisensory propositions that brands in strategic industries, such as cosmetics, offer their customers (Mensing, 2023; Spence, 2022a). Consider, for instance, the continuing growth of online shopping for cosmetics (Rodriguez et al., 2023b), where effectively conveying fragrance sensations through auditory and visual cues becomes vital in establishing product expectations, given the limitations of current digital experiences. When thinking about the impact of emotional trust, valence, and hedonic values on cosmetics online purchase intent (Suparno, 2020), brands may be motivated to use tailored soundtracks, crafting immersive experiences that deepen consumer emotional engagement. Simultaneously, traditional retail spaces may focus on delivering meaningful in-store experiences that captivate consumers through multisensory olfactory encounters. Augmenting the consumer experience across diverse channels can indeed dismantle physical barriers by intertwining the senses of smell and hearing.

Here one might also consider any synergistic effects that combining scent with matching soundscape might have in terms of enhancing the social, cognitive, or emotional impact of the former (see Spence, 2020c, 2021b). As such, should an especially good match be achieved, it remains an open question as to whether some kind of crossmodal emergent property or gestalt might be obtained. One might wonder, for instance, whether it could ever be possible to experience crossmodal harmony between the sense of scent and sound (Spence & Di Stefano, 2022; see also Velasco et al., 2023a).

Another innovative approach to the use of sound to crossmodally enhance scent/aroma came from a project with Courvoisier Cognac a decade ago, where specific timbres (e.g., harp, strings, piano) were associated with six olfactory notes (such as coffee, ginger biscuits, candied orange) that can be found in the flavour of the cognac (Crisinel et al., 2013). In this activation, customers were encouraged to smell each of the six aromas while listening to the matching sound clip. Once they had established the link between the olfactory notes and the specific matching sounds, the idea was that when they then tasted a glass of the actual cognac, they would listen to a musical composition that incorporated all of the various instrumental timbres (associated with the six specific olfactory notes) and thus that the music would help to structure and to accentuate the various olfactory notes in the temporally evolving flavor of the cognac. A similar approach has subsequently been successfully used by a number of other drinks brands (see https://www.international-sound-awards.com/overview_isa2017/nominations-isa2017/chivas-ultis/; Spence et al., 2021).

Meanwhile, research by Seo and colleagues demonstrated the crossmodal effect of sound on the perception of odor. However, in most cases, the enhancement of odor perception results from the presentation of a congruent soundscape, or else olfactory perception was shown to be impaired because of exposure to noise (Walliczek-Dworschak et al., 2016). This research capitalizes on semantic matching/congruency, that is, the associations that exist between olfaction and audition as a function of common identities or meanings (denotative meaning). For instance, in one study Seo and Hummel (2011) reported that people's enjoyment of olfactory stimuli was heightened when they were paired with semantically congruent sounds. In one case, the aroma of potato chips was combined with the sound of crunching crisps. In contrast, when incongruent sounds were presented, such as the sound of someone drinking coffee in the given scenario, the perceived pleasantness of the smell decreased (see also Seo et al., 2011, 2014; Zhou & Yamanaka, 2018).

## Crossmodal Effects of Scent on Sound

It is important to bear in mind that crossmodal effects likely also operate in the opposite direction (e.g., Guedes et al., 2023), namely from scent to people's evaluation of sound and music. In many such cases, the presentation of scent either induces a particular mood, and hence changes people's interpretation and/or assessment of music, or the scent is somehow congruent with the sound (Pizzoli et al., 2022). Several cases in which scent has been deliberately introduced to actual musical performance and those presented in literary works, have been reviewed by Spence (2021a).^21^ Meanwhile, Baccarani and Brochard (2023) recently examined whether relaxing and stimulating ambient odors (strawberry and lemon, respectively) could bias people's tempo preferences. The participants in their study listened to pairs of excerpts played at slightly different tempi and had to indicate which one they preferred. The results revealed that preferences in both odor conditions differed significantly from those in the control condition. In the absence of odor, participants preferred the slower of the fast-tempo pairs of excerpts and the faster of the slow-tempo pairs, thus indicating a general preference for moderate tempo. In the odor condition, however, the participants showed no preference for the slower of the fast-tempo pairs of excerpts in the presence of a lemon odor, and no preference for the faster of the slow-tempo pairs in the presence of a strawberry odor. Such results thus hint at the sometimes-complex nature of the crossmodal relationship between scent and sound.

## Sound and Scent in the Realm of Digital Experiences

The early stages of digitizing multisensory experiences, driven by the integration of a range of digital technologies into user and consumer interactions, gave rise to frustration and a sense of uncertainty. This likely derived from the lack of sensory cues that we traditionally depend on to navigate various offerings and offline experiences, now transferred to the online realm (Petit et al., 2019). Nevertheless, technological advances, including in virtual reality (VR), augmented reality (AR), and various other sensory-enabling technologies (Spence et al., 2017), have emerged as transformative elements capable of enhancing people's multisensory experiences involving digital technologies. As such, another area that has emerged as an intriguing field of study concerns the integration of sound and smell within such experiences mediated by digital technology, as described by Obrist et al. (2017) and Kerruish (2019) on new sensory-enabling technologies, that is, technologies that allow existing and new types of sensory stimulation and interactions in digital environments (see also Petit et al., 2019).

The integration of the olfactory and auditory senses has found interesting applications in, for example, the context of VR. For instance, Season Traveller was a VR experience developed by Ranasinghe et al. (2018) that incorporated visual, auditory, wind, thermal, and olfactory stimuli through the Samsung Gear VR Head-Mounted Display, enriching participants’ sense of presence. Here, the researchers capitalized on semantic congruence (the identity and meaning of the experience being the specific season in which they wanted the user to feel themselves immersed), thus aligning different sensory cues, including sound and smells, as a function of the four seasons. The merging of scent and sound is further illustrated in this domain by the research of Serrano et al. (2016), where VR was used to induce relaxation through the stimulation of touch and smell, but also sound. Elsewhere, meanwhile, Dozio et al. (2021) explored the use of smell and sound in visuospatial attention in VR, showing that the combination of olfactory and auditory stimuli had a greater impact on directing participants’ visuospatial attention toward the right side of the exploration space as depicted in VR. In contrast, stimuli originating from the left side led to increased performance in the left hemispace. Flavián et al. (2021) studied the impact of scent on VR experiences (including sound), with their results once again emphasizing the importance of considering aroma-content congruence. Similar recent studies were conducted to improve learning experiences using congruent scent as part VR scenes (Andonova et al., 2023), as well as in the practice of mindfulness, with scent added to immersive multisensory therapeutical atmospheres (Finck et al., 2023).

At present, many of the aforementioned experiences would appear to be focused, more than on smell and audition specifically, on the integration of multiple senses into a compelling unitary multisensory experience (or gestalt) to coincide with the corresponding storytelling. These studies, however, can also be seen as highlighting the growing importance of fusing different senses such as sound and smell to create more immersive and captivating digital experiences (see also Brengman et al., 2022).

## Conclusions

There has been an explosive growth of interest in perfumed music in recent years fueled, in part, by the literature that has emerged on the crossmodal correspondences (Deroy et al., 2013; Spence, 2011), no matter whether one is thinking about how to market scent online (Edelson, 2021; IFF, 2023), or about how to create more engaging sensory touchpoints for a brand with the consumer (Neff, 2000). It has even been suggested that scent selection can be achieved on the basis of the consumer's musical preference, perhaps hinting at the sharing of aesthetic preferences across the senses (e.g., Brown et al., 2011; Chen et al., 2022; Herz, 1998; Viengkham & Spehar, 2022). As such, there is growing agreement that one can talk meaningfully about, as well as empirically assess, the degree of congruency, or correspondence between scents and sounds (Errajaa et al., 2023; Mahdavi et al., 2020; Spangenberg et al., 2005; Zhou & Yamanaka, 2018), without having to discuss the phenomenon of synaesthesia (Anon, 2012). The evidence supporting the use of techniques such as the semantic differential technique and the emerging literature on crossmodal correspondences has been outlined. At the same time, however, the research outlined here indicates how the crossmodal congruency between sound and scent can operate at several different levels, from sematic to connotative. The many marketing-related activations in this area that have appeared over the last few years have also been reviewed.

Despite the recent hype regarding the matching of scent and sound, it remains to be seen whether this innovative new multisensory approach to the marketing and experience of scented products, such as perfume, will last in the long-term, or whether instead it is just another one of those sensory marketing fads that will pass once the headlines and media coverage have receded. However, looking to the future, it would seem possible that crossmodal correspondences between fragrance and sound might one day also be exploited in the context of sensory substitution research (Pinardi et al., 2023). For example, assuming that correspondences between olfactory fragrance notes and acoustic features of music are well-established, one could imagine conveying auditory information using odors, or *vice versa*, to allow people with impaired hearing/olfaction to experience fragrances/music more fully (i.e., in a manner that more fully engages the senses). At the same time, advances in immersive technology, such as the development of Smell-o-Vision for VR, hint at a potentially promising multisensory future in which the integration of sensory elements enhances our digital interactions (see The Verge, 2023; https://www.theverge.com/2023/5/9/23715453/wearable-smell-virtual-reality-immersive; cf. Spence, 2021d).

## Notes

1. Note that the fragrance market is expanding and was recently valued at more than $50 billion globally in 2023 (Mensing, 2023).

2. An earlier example of perfume being set to music was Harry Revel’s (1948), The “Sound” of Scents (see Anon, 2012).

3. Here, one might also consider “Essence in Space,” a project by Chang Hee Lee, based on an adapted keyboard that was directly transformed a sequence of sounds into a unique perfume via the linking of groups of musical notes with fragrances, based on Piesse’s (1867) original recommendations (see https://www.changheelee.com/essence-in-space.html).

4. According to a recent press release from IFF: “IFF today announced the launch of its first sound logo to the business-to-business (B2B) perfumery, home and personal care industries. Developed with Paris-based sound research lab IRCAM Amplify, the sound, named ‘World of Scents’, represents IFF’s creative and pioneering ambitions, and the human experience they aim to evoke” (IFF, 2023).

5. Note that in this review, we will primarily focus on the orthonasal (rather than retronasal) sense of smell, e.g., as when sniffing ambient odours (Rozin, 1982).

6. A few years ago in Germany, those customers wishing to purchase a fragrance at the perfume counter would be invited to pick a color to provide the person working at the counter with some idea as far as the customer’s likely preferences in terms of fragrance was concerned (see Spence, 2002). See also Scentmatic by Kaorium (https://www.cosmeticsbusiness.com/cosmetics-business-stand-side-with-scentmatic-209029) and Gleason-Allured (2022) for other novel approaches to the selection of personal scent.

7. Such an approach can easily be based on tracking a consumer’s musical preferences (e.g. Youtube, Spotify) and then matching them with “fragrance.”

8. Here, it is worth noting that ISO 12913-1:2014 (Acoustics — Soundscape — Part 1: Definition and conceptual framework) provides a particular definition and a conceptual framework for thinking about the notion of a “soundscape.” According to the definition, a distinction can be drawn between the acoustic environment and the soundscape, where the latter is considered to be the perception of the former, in context. As such, the reproduction of sound from a loudspeaker would refer to the concept of an acoustic environment, while the auditory perception of that sound would refer to the concept of the soundscape. It should, however, be noted that the term “soundscape” has different meanings within different communities of researchers (e.g., Grinfeder et al., 2022; Schafer, 1977; Southworth, 1969). Here, the term is used in the way it is defined in the *Cambridge Dictionary*, namely: “A work of art or performance that combines sounds in order to create a particular effect.”

9. Here, it may be helpful to distinguish between connotative and denotative meaning. Denotative meaning is the direct, agreed-upon definition of a word, linking it to specific real-world things or concepts, whereas connotative meaning is the emotional or cultural associations a word evokes in a person, beyond its direct, dictionary definition (Osgood, 1959).

10. The latter, a rare condition whereby a small percentage of the population experiences a concurrent sensory experience in one sense, is based on stimulation that is often presented in another (see Ward, 2013, for a review).

11. “There are a lot of bridges between perfumery and music,” said Jean-Christophe Hérault, an IFF master perfumer who worked on Spicebomb with perfumer Carlos Benaim (as quoted in Edelson, 2021).

12. Listen to the sonic logo at https://iff.showpad.com/share/F6HYrnllRohjUvqJGYNVE. It would obviously be important to see whether such marketing claims stand up to a rigorous peer-review process.

13. In this collaboration, Ephemera by Unsound, a fragrance project from Unsound Productions (of the Unsound Festival) and perfumer Geza Schoen, three limited edition fragrances were released (Noise, Drone, and Bass). The fragrances were inspired by musical material that had been created by three musicians—Ben Frost, Tim Hecker, and Steve Goodman (aka Kode9) (see Robin, 2015).

14. According to the press release (8^th^ Dec 2007: Vivere in tune with the senses): Latin American consumers buy into fragrance: Vivere fabric conditioners in Argentina and Uruguay have used music and longer-lasting fragrance range to increase sales.

15. One of the key problems here is the idiosyncratic nature of the inducer-concurrent relation in the case of synaesthesia (Spence, 2012, 2015a). This means that it is difficult to ascertain what consensual crossmodal mappings might be. Furthermore, it should also be noted that there are very few synaesthetes who report experiencing sound-scent synaesthesia.

16. This likely relates with the shared feature of both sounds and odors, namely, the fact that they are essentially identified, and allegedly perceived, as linked to their source.

17. These aromas are strongly linked with a sour and sweet taste, respectively.

18. It is interesting here to note the instruction to avoid such direct semantic matches, such as the smell of coffee being associated with the coffee machine (e.g., Seo et al., 2011, 2014; Seo & Hummel, 2011). While those crossmodal correspondences that are based on an individual’s semantic knowledge undoubtedly constitute legitimate ways of connecting the senses of hearing and olfaction, such links are perhaps too obvious to necessarily excite the curiosity of the creative composer or perfumer. Furthermore, it is worth noting how the majority of olfactory sources in the environment have no specific sonic attributes (e.g., it is hard to think of a semantically matching sound for the scent of a rose, say), nor do musical instruments (such as the violin or trumpet) have specific olfactory attributes attached.

19. The synthesized timbres that were matched to their intended scent (e.g., as expected based on the intuitions of the composer) were lemon, lemon blossom, melon, vanilla, and caramel. Additionally successful, although not with intended scents (partly because many of them were lacking from the aromatic stimulus set), were [flowers] with the lemon blossom, melon, and cherry scents, [truffle] with the tobacco scent, [tobacco] with the coffee scent, [bergamot], [sour], [cocoa], and [coffee] with the pepper scent, [strawberry] with the lemon scent, and [blackberry] with the lemon blossom scent.

20. See https://soundcloud.com/xmodal/sets/sweetdry, for some of the examples used by Velasco and Spence (2022).

21. For example, in Huxley’s *Brave New World* (1932, pp. 198–199), the scent organ is presented: “…The scent organ was playing a delightfully refreshing Herbal Capriccio—rippling arpeggios of thyme and lavender, of rosemary, basil, myrtle, tarragon; a series of daring modulations through the spice keys into ambergris: and a slow return through sandalwood, camphor, cedar and new-mown hay (with occasional subtle touches of discord—a whiff of kidney pudding, the faintest suspicion of pig dung) back to the simple aromatics with which the piece began.”
